# 29120 Classification of Individuals Across the Spectrum of Problematic Opioid Use: Clinical Correlates and Longitudinal Associations with Mortality

**DOI:** 10.1017/cts.2021.753

**Published:** 2021-03-30

**Authors:** Victoria Powell, Colin MacLeod, Lewei A. Lin, Amy S.B. Bohnert, Pooja Lagisetty

**Affiliations:** 1 University of Michigan Division Geriatric and Palliative Medicine and VA Ann Arbor Healthcare System Geriatric Research, Education and Clinical Center; 2 University of Michigan Department of Internal Medicine and Center for Clinical Management and Research, Ann Arbor VA Hospital; 3 University of Michigan Department of Psychiatry and Center for Clinical Management and Research, Ann Arbor VA Hospital; 4 University of Michigan Department of Psychiatry and Department of Anesthesiology, Center for Clinical Management and Research, Ann Arbor VA Hospital

## Abstract

ABSTRACT IMPACT: A better understanding of the spectrum of problematic opioid use will lead to more targeted treatments. OBJECTIVES/GOALS: It is unclear how to approach treatment of individuals with problematic opioid use who do not clearly meet criteria for opioid use disorder (OUD). We aim to characterize clinical, demographic, and medication use at time of identification of problematic opioid use across the spectrum as well as identify predictors of poor outcomes. METHODS/STUDY POPULATION: A national sample of Veterans coded as having opioid abuse or dependence were previously categorized as (1) high likelihood of OUD, (2) limited aberrant opioid use, and (3) prescribed opioid use with no evidence of aberrant use based on chart review. We will describe how individuals in these three categories differ demographically and clinically. We will then use a trained binary logistic regression model to predict whether individuals with limited aberrant opioid use more closely resemble category (1) or (3). Cox proportional hazards models will be used to predict all-cause mortality, suicide-related mortality, opioid-overdose related mortality, and hospitalization over a three-year period using the three categories as predictors and adjusting for relevant covariates. RESULTS/ANTICIPATED RESULTS: We anticipate that Veterans with a high likelihood of OUD will be more likely to experience homelessness and have more psychiatric comorbidities (especially PTSD). We hypothesize that Veterans with prescribed opioid use and no evidence of misuse will be significantly older, more likely to have disability, medical comorbidities (ie., chronic pain, cancer), more prescriptions for non-opioid analgesics, and be prescribed higher doses of opioids. Using a trained binary logistic regression model, we predict that Veterans with limited aberrant opioid use will more closely resemble Veterans with a high likelihood of OUD. We expect that all categories of problematic opioid use will have a high risk of mortality, with a high likelihood of OUD associated with the greatest risk of premature death. DISCUSSION/SIGNIFICANCE OF FINDINGS: Identifying and better characterizing individuals with limited aberrant opioid use may be an important opportunity to intervene prior to development of severe OUD. Future research will focus on targeting interventions to this population, which may have specific needs that are separate from classic OUD or simple pain-related opioid dependence.

